# Inhibition of Connexin 43 and Phosphorylated NR2B in Spinal Astrocytes Attenuates Bone Cancer Pain in Mice

**DOI:** 10.3389/fncel.2018.00129

**Published:** 2018-05-08

**Authors:** Hui Yang, Hui Yan, Xin Li, Jing Liu, Shousong Cao, Baisheng Huang, Dong Huang, Lixiang Wu

**Affiliations:** ^1^Department of Anesthesiology, The Third Xiangya Hospital, Central South University, Changsha, China; ^2^Department of Physiology, School of Basic Medical Science, Central South University, Changsha, China; ^3^Department of Pain, The Third Xiangya Hospital and Institute of Pain Medicine, Central South University, Changsha, China; ^4^Department of Neurosurgery, The Third Xiangya Hospital, Central South University, Changsha, China; ^5^Department of Pharmacology, School of Pharmacy, Southwest Medical University, Luzhou, China

**Keywords:** bone cancer pain, spinal dorsal horn, astrocyte, connexin 43, NR2B, phosphorylation, NMDA receptors, mice

## Abstract

Bone cancer pain (BCP) is common in patients with advanced cancers when the tumors are metastasized to bone. The limited understanding of the complex pathogenesis of BCP leads to the poor effectiveness of clinical treatment. Previous studies have shown that astrocyte-specific connexin (Cx) 43, a forming protein of gap junction (GJ) and hemichannel, and N-methyl-D-aspartate receptors (NMDARs), especially the phosphorylated NMDAR 2B subunit (NR2B) phosphorylated NR2B (p-NR2B) subunit are involved in BCP. However, the relationship between Cx43 and p-NR2B in BCP remains unclear. In the present study, we investigated the expressions of Cx43, glial fibrillary acidic protein (GFAP, a marker of astrocytes), and p-NR2B in the spinal dorsal horn (SDH) in a mouse model of BCP established by intra-femural inoculation of Lewis lung carcinoma (LLC) cells via intrathecal (ith) injection of the GJ/hemichannel blocker carbenoxolone (CARB) and the NMDAR antagonist MK801, respectively. We found that the characters of BCP were mimicked by intra-femural inoculation of LLC cells in mice, and the expressions of Cx43, GFAP and p-NR2B in BCP mice were remarkably increased in a time-dependent manner from day 7 to day 21 after cell inoculation with a gradual aggravate in spontaneous pain and mechanical allodynia. Furthermore, Cx43 was predominantly expressed in the spinal astrocytes. Both CARB and MK801 inhibited the expressions of Cx43, GFAP and p-NR2B with attenuated pain hypersensitivity in BCP mice. In addition, Cx43 was co-localized with p-NR2B in the SDH, which further evidenced the presence of functional NR2B in the spinal astrocytes in BCP mice. Our findings demonstrate that inhibition of Cx43 and p-NR2B in spinal astrocytes could attenuate BCP in mice and Cx43 and p-NR2B in the astrocytes of the SDH may play an important role via their combination action in the development and maintenance of BCP in mice. These results may provide a potential therapeutic target in the prevention and/or treatment of BCP.

## Introduction

Bone cancer pain (BCP) is common in patients with advanced cancers, and approximately 75% of cancer patients at advanced stage experienced moderate and severe BCP, which severely affected their daily activities and quality of life (Falk and Dickenson, 2014; Krzeszinski and Wan, 2015). Many common cancers such as breast, lung, kidney and prostate have a propensity to metastasize to various bones of the body including the hip, vertebrae, femur, tibia and ribs. Thus, patients with BCP generally exhibit radiographic evidence of cancer-induced bone destruction or fracture (Mantyh, 2014). Patients with BCP often suffer from a complex pain including a combination of background pain (a dull continuous pain, gradually increasing in intensity with time), spontaneous pain (without a clear stimulus), and evoked pain (caused by a stimulus, commonly movement; Currie et al., 2013). Currently, the “World Health Organization (WHO) analgesic ladder” remains the main clinical therapy for the management of the patients with BCP but it often fails to effectively control the pain due to inadequate analgesia and/or intolerable side effects (Mantyh, 2013). However, BCP are complex and mixed with neuropathic, inflammatory, ischemic and tumor-specific mechanisms. The characteristics of BCP are involved in the elements of both neuropathic pain and inflammatory pain with a unique neurochemical change in the spinal cord (Goblirsch et al., 2005). Therefore, it is vital to investigate the underlying cellular and molecular mechanisms of BCP and discover feasible targets for development of more effective novel pharmacological therapy.

Astrocytes are the major glial cells widely distributed throughout the central nervous system (CNS) and have been traditionally considered as just providing trophic, structural and metabolic supportive role for neuronal function (Pellerin et al., 2007). Astrocytes are crucial for maintaining the homeostasis of the microenvironment of neurovascular unit and CNS with close involvement in neuronal development, regeneration, synaptic transmission, immune and spatial buffering of various signaling molecules (Salmina et al., 2014; Liddelow and Barres, 2017). However, recent studies have demonstrated that astrocytes actively participate in synaptic activity and contribute to maintain pathological pain via interaction with neurons (Ren, 2010; Chever et al., 2014). An important feature of astrocytes is that the cells communicate with each other via wide networks formed by gap junction (GJ) channels. The astroglial networks modulate synaptic transmission and plasticity, and subsequently regulate neuronal networks activity (Pannasch and Rouach, 2013). However, it remains elusive for how is the action of astrocyte-astrocyte signaling of astroglial networks exerted in BCP.

Recently, accumulating evidence has demonstrated that Connexin (Cx) 43 plays a significant role in many pathological conditions including Alzheimer’s disease (Yi et al., 2016), amyotrophic lateral sclerosis (Almad et al., 2016), hypoxic/reoxygenation injury (Vicario et al., 2017a), retinal diseases (Danesh-Meyer et al., 2016) and obesity (Chen et al., 2015), In the CNS, Cx43 is a transmembrane protein mainly expressed by astrocytes to constitute GJ or hemichannel to regulate electric and metabolic activities between neighboring astrocytes or intracellular and extracellular astrocyte, and subsequently maintains the homeostasis of the extracellular environment of neurons (Charveriat et al., 2017). It is well established that astrocyte-specific Cx43 was involved in neuropathic pain and morphine antinociceptive tolerance in various animal models (Chen et al., 2012, 2014; Yoon et al., 2013; Shen et al., 2014; Xu et al., 2014; Robinson and Dougherty, 2015; Choi et al., 2016). It has been reported that phosphorylated Cx43 (p-Cx43) mediated chemokine CXCL12 production from spinal astrocytes to maintain BCP in rats (Hang et al., 2016). The referred studies may demonstrate the role of Cx43-CXCL12-CXCR4 pathway in astrocyte-neuron signaling in central sensitization for maintenance of BCP, however, the role of Cx43-related astrocyte-astrocyte signaling in BCP remains unclear.

Studies have indicated that N-methyl-D-aspartate receptor 2B subunit (NR2B; especially phosphorylated NR2B p-NR2B), one of the subunits of N-methyl-D-aspartate receptor (NMDAR), participates in central sensitization and plays a crucial role in various pain, including neuropathic pain (Kim et al., 2012), inflammatory pain (Bu et al., 2015) and BCP (Liu et al., 2015; Sun et al., 2016). A previous study has demonstrated that GJ blocker carbenoxolone (CARB) attenuated neuropathic pain by suppressing NMDAR activation (Roh et al., 2010). Moreover, it was considered that the interaction between astrocytic Cx43 and neuronal NMDARs may represent the significant function of astrocytes-neurons signaling in the spinal cord (Shen et al., 2014). However, NR2B, traditionally known as neuron-specific, has been found expressed in primary astrocytes including spinal astrocytes of BCP rats (Krebs et al., 2003; Liu et al., 2013). Therefore, we speculate that the combination action of Cx43 and p-NR2B in astrocytes of the spinal dorsal horn (SDH) may contribute to the development and maintenance of BCP in mice, and the combination action between spinal Cx43 and astrocytic p-NR2B may reflect the function of astrocyte-astrocyte signaling in BCP.

To confirm this hypothesis, in the present study, we investigated the expressions of Cx43, glial fibrillary acidic protein (GFAP, a marker of astrocytes), and p-NR2B in the SDH and assessed pain-related behaviors in a mouse model of BCP. Furthermore, the role of Cx43 and p-NR2B in BCP was studied via intrathecal (ith) injection of the GJ/hemichannel blocker CARB and the NMDAR antagonist MK801 and co-localization of Cx43 and p-NR2B in the SDH of BCP mice.

## Materials and Methods

### Cell Culture

Lewis lung carcinoma (LLC) cell line (murine lung adenocarcinoma cell), was purchased from the Cell Bank of Xiangya School of Medicine of Central South University (Changsha, Hunan, China). LLC cells were cultured in Dulbecco’s modified Eagle medium (DMEM; Gibco, Gaithersburg, MD, USA) containing 25 mM glucose supplemented with 10% fetal bovine serum (FBS; Gibco, Gaithersburg, MD, USA), 1% penicillin/streptomycin (Gibco, Gaithersburg, MD, USA) at 37°C in a humidified atmosphere of 5% CO_2_ and 95% O_2_. The cells were passaged every 2 days at a ratio of 1:2.

### Experimental Animals

Specific-pathogen-free (SPF) grade male C57BL/6 mice (6–8 week-old, body weight 20–25 g) were purchased from Hunan Silaikejingda (SJA) Laboratory Animal Co., Ltd (Changsha, Hunan, China). All animals were housed up to five per cage with *ad libitum* access to water and food in a light-controlled (12/12 h light/dark cycle) and temperature-controlled (22 ± 0.5°C) room. All procedures in this study were complied with the ethical guidelines of the International Association for the Study of Pain (Zimmermann, 1983) and were approved (protocol No. LLSC(LA) 2016–017) by the Committee on Animal Care and Use of The Third Xiangya Hospital of Central South University (Changsha, Hunan, China). All efforts have been made to decrease the use of number of experimental animals and to minimize the animal suffering.

### Surgical Procedure for Establishing a Mouse Model of BCP

LLC cells were detached from the flask by 0.25% trypsin and 0.02% Ethylenediaminetetraacetic Acid (EDTA; Gibco, Gaithersburg, MD, USA) for subsequent preparation of injection. Briefly, the cells were first collected by centrifugation of 10 ml of cell suspension at 106× *g* for 5 min. The pellet was resuspended in Hank’s balanced salt solution (HBSS) and the cells were counted with a TC10^™^ automated cell counter (Bio-Rad, Hercules, CA, USA). Then, the cells were diluted with HBSS (Gibco, Gaithersburg, MD, USA) at a final concentration of 2 × 10^5^ cells/μl for inoculation. The cells were injected into the intramedullary space of femur according to the modified method reported previously (Huang et al., 2008; Isono et al., 2011). Mice were kept at supine position after anesthetized with intraperitoneal (i.p.) of 2% pentobarbital sodium (50 mg/kg). The left leg of mouse was shaved and a superficial incision (0.5 cm) was made in the skin overlying the condyles of the distal femur after applying with fortified iodine solution for disinfection. Then a blunt dissection was performed on the patellar ligament to expose the condyles of the distal femur with minimal damage. 10 μl of LLC cell suspension (2 × 10^5^ cells/μl) was slowly injected into the medullary cavity of the left distal femur using a 25 μl sterile microsyringe. The syringe was left in place for 90 s to allow cells to fill the bone cavity and the injection hole was sealed with sterile bone wax (Braun, Rubi, Barcelona, Spain) to prevent cell leakage after removal of the syringe. The skin was then stitched with 3–0 silk threads to close the wound and smeared with chlorotetracycline ointment (Baijingyu Pharmaceutical Co. LTD, Nanjing, Jiangsu, China). Control mice were injected with 10 μl of sterile HBSS instead of LLC cells. The general health condition and body weights of control and BCP mice were monitored on day 0 (baseline, prior to inoculation), 7, 14 and 21 after cell inoculation.

### Drugs Administration

CARB (Sigma-Aldrich, St. Louis, MO, USA) and MK801 (Sigma-Aldrich, St. Louis, MO, USA) were dissolved in normal saline (NS, 0.9% sodium chloride) at the concentrations of 2.5 and 0.68 μg/μl, respectively. CARB (1 mg/kg, 25 μg in 10 μl) was administered by ith injection once a day for 14 days (on day 7–20 postoperatively). MK801 (0.136 mg/kg, 3.4 μg in 5 μl) was administered by ith injection once a day for 10 days (on day 11–20 postoperatively). Drug administration was performed on animals with a 10 μl Hamilton syringe attached to a 30-gauge needle as previously described with the following modifications (Hylden and Wilcox, 1980). Briefly, the mouse was tightly held by the middle finger and thumb at the level of bilateral iliac crests, and the fifth lumbar (L_5_) spinous process was verified by palpating the highest spinous process with the index finger. The needle was inserted into the L_5_-L_6_ intervertebral space and the successful insertion of the needle into the lumbar subarachnoid space was identified by a tail flick response. Each drug was slowly injected for more than 20 s. Then the needle was left in place for an additional 20 s before removal. The control mice were received an equivolume injection of NS. The dose selection for CARB and MK801 was based on the results from our previous experiments and reports from the literatures (Yoon et al., 2013; Shen et al., 2014; Uchida et al., 2014). Ten mice were used for each experimental group.

### Pain Behavioral Tests

The mice were examined for changes in spontaneous foot-lifting (SFL) behaviors, the indication of spontaneous pain as described previously (Ren et al., 2012). SFL was defined as a lift of the hind paw not correlated with grooming or walking. To assess the SFL behaviors, mice were placed in a transparent acrylic box put on a horizontal surface, and allowed them to habituate for 20 min before the test to record the cumulative duration of SFL for 4 min. The tests were performed twice for each mouse with a test interval of 5 min. The results were calculated from the average of two tests.

In addition, mechanical allodynia was determined by paw withdrawal mechanical threshold (PWMT) in response to an ascending series of Von Frey hair stimulation, as previously described (Yoon et al., 2013). In brief, mouse was placed in a 26 cm × 14 cm × 26 cm Plexiglas box equipped with a metallic mesh floor, 22 cm above the bench and allowed it to acclimate for 20 min before the test. Each Von Frey hair (North Coast Medical, Gilroy, CA, USA) was applied six times in ascending order to the mid-plantar surface of each hind paw of mouse, and held for 3–4 s with a 2 min interval between tests. The lowest Von Frey hair that produced a paw withdrawal, flinch, or lick in three of the six applications was regarded as the 50% paw withdrawal threshold. All the behavioral tests were performed at the same time in the morning to avoid different influence on mice from the variation of circadian rhythm and other activities and by the same experimenter. The pain behaviors were observed on day 0 (baseline, before cell inoculation), 7, 14, and 21; or on day 0 (baseline, before cell inoculation), 11, 14, 16, 19, and 21 after cell inoculation for the experiments of MK801. Ten mice were used for each experimental group.

### Bone Evaluation in Control and BCP Mice by Radiology and Histology

Mice were anesthetized with 2% pentobarbital (150 mg/kg, i.p.), and sacrificed by decapitation on day 21 after LLC cells or culture medium (HBSS) inoculation. Then, the bilateral hind limbs were cut from the body of mice to obtain the images of bilateral hind limbs of control and BCP mice by radiographs (intensity with 10-mAs, and voltage 30-kV, Senographe Essential, GE Healthcare, Boston, MA, USA). For pathohistological evaluation, the bilateral femurs with muscles were removed and fixed in 4% paraformaldehyde overnight at 4°C, then demineralized in decalcifying solution for 24 h. The tissues were embedded in paraffin after rinsed and dehydrated, cut into 4-μm thick sections with a rotary microtome (Leica RM2235; Leica Microsystems, Heidelberg, Baden-Wurttemberg, Germany), and stained with hematoxylin and eosin (HE) to observe the tumor cells infiltration and bone destruction in control and BCP mice under a microscope. Five mice were used for each experimental group.

### Western Blotting

Mice were sacrificed by decapitation after anesthetized with pentobarbital (150 mg/kg, i.p.). The L_4_-L_5_ spinal cord segments were quickly removed from the spine after laminectomy and stored at −80°C until further processing. The tissues were homogenized in a tissue protein extraction reagent buffer containing protease and phosphatase inhibitors (Sigma-Aldrich, St. Louis, MO, USA). Then homogenates were centrifuged at 13,400× g for 15 min at 4°C. The supernatants were collected for assessment of protein concentration by Bicinchoninic Acid (BCA) Protein Assay method. Then equivalent amounts of protein (40 μg) were separated on 10% SDS-PAGE gel (Bio-Rad, Hercules, CA, USA), and transferred onto polyvinylidene difluoride filters (PVDF; Millipore, Bedford, MA, USA) to form blots. The protein blots were subsequently blocked in blocking solution (5% non-fat milk in TBS with 0.1% Tween-20) for 1 h and then incubated with Cx43 rabbit polyclonal antibody (1:2000, cat# C6219, Sigma-Aldrich, St. Louis, MO, USA), GFAP mouse monoclonal antibody (1:1500, cat# 3670, Cell Signaling Technology, Beverly, MA, USA), and p-NR2B rabbit polyclonal antibody (1:250, cat# M2442, Sigma-Aldrich, St. Louis, MO, USA) at 4°C for overnight, followed by incubation with horseradish peroxidase (HRP)-conjugated goat anti-rabbit or goat anti-mouse secondary antibody (1:2000, cat# CW0103 and CW0110S, Cwbiotech, Beijing, China) at room temperature for 2 h. Bands were visualized using Enhanced Chemiluminescence System (GE Amersham Biosciences, Boston, MA, USA) and exposed onto film for 1–5 min. For loading control, blots were further stripped and re-probed with glyceraldehyde-3-phosphate dehydrogenase (GAPDH) rabbit antibody (1:2000, cat# 10494-1-AP, Proteintech, Chicago, IL, USA) followed by HRP-conjugated goat anti-rabbit secondary antibody (1:2000, cat# CW0103, Cwbiotech, Beijing, China). The intensity of the specific bands was captured and analyzed using ImageJ software (NIH, Bethesda, MD, USA). Five mice were used for each experimental group.

### Immunohistochemistry and Immunofluorescence

Mice were perfused transcardially with 0.9% NS followed by 4% paraformaldehyde in 0.1 M phosphate buffer saline (PBS, pH 7.4) after anesthetized with 2% pentobarbital (150 mg/kg, i.p.). The L_4_-L_5_ spinal cords were removed, post-fixed in 4% paraformaldehyde for overnight at 4°C. The spinal cords were then cryoprotected by immersion in 15% sucrose, dissolved in 0.1 M PBS (pH 7.4), and followed by in 30% sucrose until sectioning. The embedded blocks were sectioned as 20 μm thick in a cryostat (Leica CM1950; Leica Microsystems, Heidelberg, Baden-Wurttemberg, Germany) and stored in PBS for immunofluorescence. Briefly, the sections were blocked with 5% donkey serum (Jackson ImmunoResearch Europe Ltd., Cambridge, Cambs, UK) in 0.01 M PBS (pH 7.4) with 0.3% Triton X-100 for 1 h at room temperature and incubated overnight at 4°C with primary antibodies in PBS with 0.3% Triton X-100: Cx43 goat polyclonal antibody (1:300, cat# LS-B9771, LSBio, Seattle, WA, USA), GFAP mouse monoclonal antibody (1:300, cat# 3670, Cell Signaling Technology, Beverly, MA, USA), and p-NR2B rabbit polyclonal antibody (1:50, cat# M2442, Sigma-Aldrich, St. Louis, MO, USA), respectively. The sections were rinsed with PBS for three times, then incubated with the DyLight^™^ 488 or 594 conjugated secondary antibodies (1:500, cat# 123864, 123589 and 122684, Jackson ImmunoResearch Europe Ltd., Cambridge, Cambs, UK) for 2 h at room temperature. The sections were washed three times with PBS, and then mounted with mounting medium on the glass slides. For double staining, the sections were incubated with a mixture of primary antibodies: Cx43 goat polyclonal antibody (1:300, cat# LS-B9771, LSBio, Seattle, WA, USA) with GFAP mouse monoclonal antibody (1:300, cat# 3670, Cell Signaling Technology, Beverly, MA, USA), NeuN rabbit polyclonal antibody (1:500, cat# ab104225, Abcam, Cambridge, Cambs, UK), Iba1 rabbit polyclonal antibody (1:500, cat# 019-19741, Wako, Osaka, Osaka Prefecture, Japan), or p-NR2B rabbit polyclonal antibody (1:50, cat# M2442, Sigma-Aldrich, St. Louis, MO, USA), respectively, and then followed by a mixture of the DyLight^™^ 488 or 594 conjugated secondary antibodies (1:500, cat# 123864, 123589 and 122684, Jackson ImmunoResearch Europe Ltd., Cambridge, Cambs, UK). Finally, the stained sections were examined under a fluorescence microscopy (Nikon ECLIPSE 80i, Tokyo, Japan), and the images were captured with a Nikon Digital Sight. The mean fluorescence intensity of specific staining was calculated and analyzed using Image-Pro Plus software (Media Cybernetics, Inc., Rockville, MD, USA). Double immunofluorescent staining images were obtained with a confocal microscope (Leica, TCS SP8 X&MP, Heidelberg, Baden-Wurttemberg, Germany). Five mice were used for each experimental group.

### Statistical Analysis

Data were analyzed by IBM SPSS statistics software version 19.0 (IBM Corp., Armonk, NY, USA) and presented as mean ± standard error (SE). Graphs were created with GraphPad Prism version 6.02 (GraphPad Software, San Diego, CA, USA). Intergroup comparisons of the data of body weights and behavioral tests were evaluated with two-way analysis of variance (ANOVA), and comparisons between multiple time points were examined with repeated-measures ANOVA. The data of Western blotting and immunohistochemistry were compared with one-way ANOVA followed by Tukey’s test. Differences between two groups were also compared by Student’s *t*-test. *p* < 0.05 was considered to be statistically significant (marked as *) and the higher significance level was set at *p* < 0.01 (marked as **) or *p* < 0.001 (marked as ***).

## Results

### Intra-Femural Inoculation of LLC Cells Mimics the Characters of BCP in Mice

**Figure 1:**
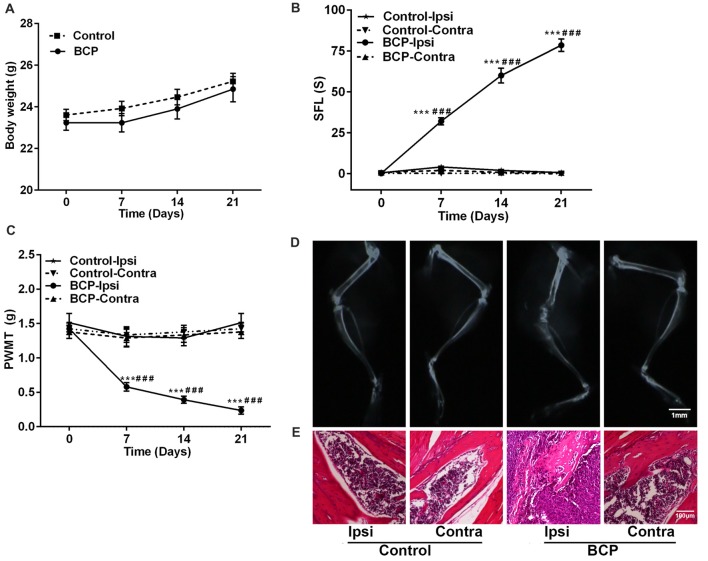
Intra-femural inoculation of Lewis lung carcinoma (LLC) cells mimics the characteristics of bone cancer pain (BCP) in mice. **(A)** Body weights were increased in both control and BCP mice. **(B)** Evaluation of the ipsilateral spontaneous foot-lifting (SFL) in control and BCP mice. **(C)** Evaluation of the ipsilateral paw withdrawal mechanical threshold (PWMT) in control and BCP mice. **(A–C)** Evaluations were performed on day 0 (control, baseline, before cell inoculation), 7, 14 and 21 after cell inoculation. Ten mice were used for each group (*n* = 10). Data were presented as mean ± standard error (SE), ***p < 0.001 vs. control mice. ^###^*p* < 0.001 vs. baseline (day 0). **(D)** Evaluation of the ipsilateral distal femurs in normal and BCP mice on day 21 after medium solution or cell inoculation by radiation analysis. Bar = 1 mm. **(E)** Evaluation of the ipsilateral distal femurs in normal and BCP mice on day 21 after medium solution or cell inoculation by pathohistological analysis. Bar = 100 μm. **(D,E)** Five mice were used for each group (*n* = 5).

After LLC cells were injected into the intramedullary space of the femur, the mice were in general good health with a tendency of body weight gain. There was no significant difference in body weight changes between the mice injected with medium solution (control) and the mice inoculated with LLC cells during a 21-day observation period (Figure 1A). However, LLC cells inoculated into the femur induced remarkable pain including spontaneous pain and mechanical allodynia in the ipsilateral hind paws of BCP mice. The cumulative duration was significantly increased from 0.22 ± 0.05 s before cell inoculation (on day 0) to 32.37 ± 5.84 s, 60.71 ± 16.32 s, and 78.44 ± 11.42 s on day 7, 14 and 21, respectively after inoculation (*p* < 0.001) by SFL behavior test (Figure 1B). Furthermore, the ipsilateral hind PWMT was decreased from 1.42 ± 0.45 g before cell inoculation (on day 0) to 0.58 ± 0.17 g, 0.39 ± 0.12 g and 0.24 ± 0.14 g on day 7, 14 and 21, respectively after inoculation (*p* < 0.001, Figure 1C). The results indicate a gradual development of mechanical allodynia in the ipsilateral hind paws of BCP mice. However, no significant change of pain was observed in the contralateral hind paws of BCP mice or bilateral hind paws of control mice (Figures 1B,C).

Moreover, the ipsilateral distal femurs of BCP mice were suffered from pathological fracture accompanied by the destruction of medullary bone and cortical bone with swelling and distension of peripheral tissues on day 21 after cell inoculation by X-ray examination. The ipsilateral distal femurs of BCP mice also showed that tumor cells were packed in the medullary cavity and invaded into the peripheral tissues via penetrating through the damaged cortical bone by pathohistological analysis. However, no change was found in the contralateral femurs of BCP mice or bilateral femurs of control mice by radiological or histological examinations (Figures 1D,E).

### BCP Mice Exhibit Increased Levels of Cx43 and GFAP in the SDH

**Figure 2:**
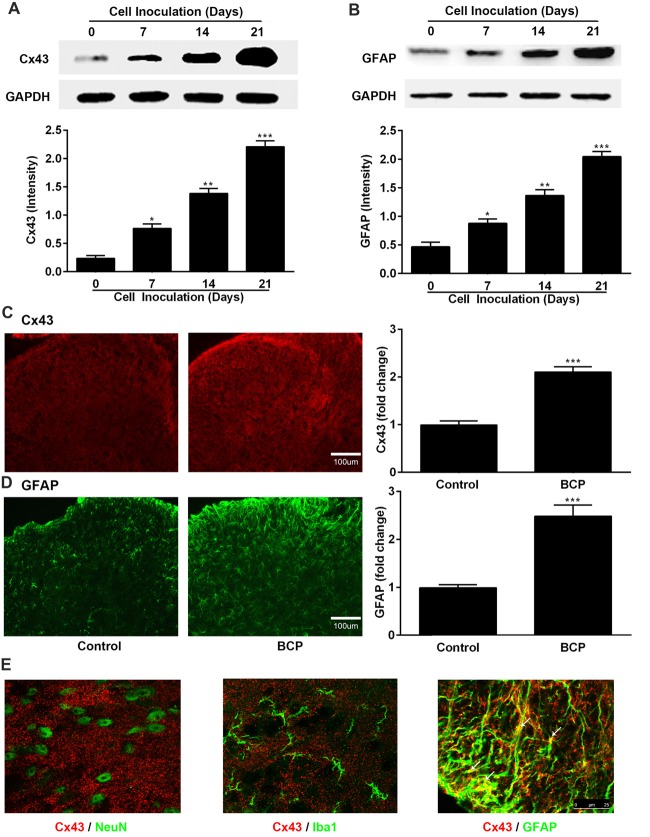
The expressions of Cx43 and glial fibrillary acidic protein (GFAP) in the spinal dorsal horn (SDH) in normal and BCP mice. **(A)** Representative bands and summarized data of expression of Cx43 on day 0 (control, baseline, before cell inoculation), 7, 14, 21 after cell inoculation by Western blotting analysis. **(B)** Representative bands and summarized data of expression of GFAP on day 0 (control, baseline, before cell inoculation), 7, 14, 21 after cell inoculation by Western blotting analysis; glyceraldehyde-3-phosphate dehydrogenase (GAPDH) was used as a loading control for Western blotting analysis. **(C)** Representative images and summarized data of Cx43 on day 21 after cell inoculation by immunofluorescence analysis. **(D)** Representative images and summarized data of GFAP on day 21 after cell inoculation by immunofluorescence analysis; Bar = 100 μm. **(E)** Confocal images of co-localization of Cx43 with GFAP (astrocytic marker), with NeuN (neuronal marker), or with Iba1 (microglial marker) in the SDH in BCP mice by double staining. Bar = 25 μm. **(C–E)** Tissues of spinal cord were collected from control and BCP mice on day 21 after cell inoculation. There were five mice used for each group (*n* = 5). Data were presented as mean ± SE. *p < 0.05, **p < 0.01, ***p < 0.001 vs. control mice.

Next, we investigated the expressions of Cx43 and GFAP in the SDH in BCP mice on day 0, 7, 14 and 21 after inoculation of LLC cells by Western blotting analysis and the results are shown in Figures 2A,B. The expression of Cx43 in the SDH was remarkably elevated in a time-dependent manner compared to that of control mice (on day 0). The increased expression of Cx43 was initiated on day 7 (*p* < 0.05), remained higher level on day 14 (*p* < 0.01), and peaked at day 21 (*p* < 0.001), the last day of experiment. In addition, the expression of GFAP was also remarkably upregulated similar to that of Cx43, indicating that the astrocytes were activated in BCP (Figures 2A,B).

Similarly, the Cx43-immunoreactivity (IR) and GFAP-immunoreactivity (IR) were also upregulated in the ipsilateral SDH of BCP mice on day 21 after cell inoculation, compared to that of control mice (*p* < 0.001) by immunostaining results (Figures 2C,D). Moreover, Cx43 in the SDH was extensively co-localized with GFAP (astrocytic marker), but not with NeuN (neuronal marker) or Iba1 (microglial marker) by confocal image analysis from double immunofluorescent staining in the spinal cord sections of BCP mice (Figure 2E), suggesting that Cx43 was expressed in astrocytes, but not in neurons or microglial cells.

### Increased Expression of p-NR2B in the SDH in BCP Mice

**Figure 3:**
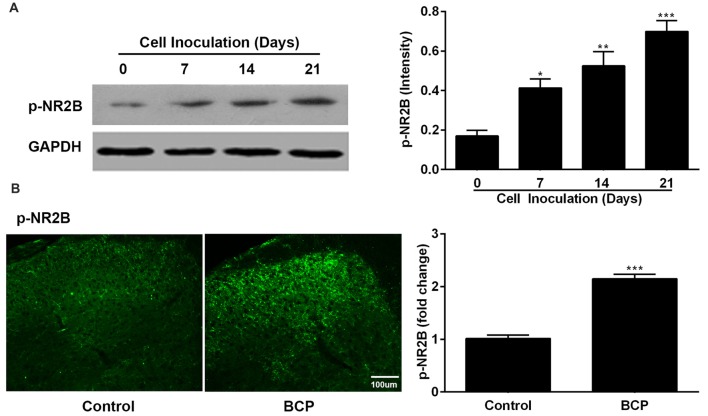
The expression of phosphorylated NR2B (p-NR2B) in the SDH in control and BCP mice by Western blotting analysis **(A)** and immunofluorescence analysis **(B)**. Western blotting analysis was performed on day 0 (control, baseline, before cell inoculation), 7, 14, 21 and immunofluorescence analysis was performed on day 21 (Bar = 100 μm) after cell inoculation. GAPDH was used as a loading control for Western blotting analysis. There were five mice used for each group (*n* = 5). Data were presented as mean ± SE. *p < 0.05, **p < 0.01, ***p < 0.001 vs. control mice.

In parallel, we also examined the expression of p-NR2B in the SDH in BCP mice on day 0, 7, 14 and 21 after LLC cells inoculation. The protein level of p-NR2B was persistently increased on 7 (*p* < 0.05), 14 (*p* < 0.01), and 21 (*p* < 0.001) days after inoculation with LLC cells in BCP mice compared to that of control mice (on day 0) by Western blotting analysis (Figure 3A). Furthermore, the immunoreactivity of p-NR2B was also significantly increased in the ipsilateral SDH of BCP mice on day 21 after cell inoculation compared to that of control mice (*p* < 0.001) by immunostaining analysis (Figure 3B).

### CARB Attenuates BCP and Suppresses the Expressions of Cx43 and p-NR2B in the SDH in BCP Mice

**Figure 4:**
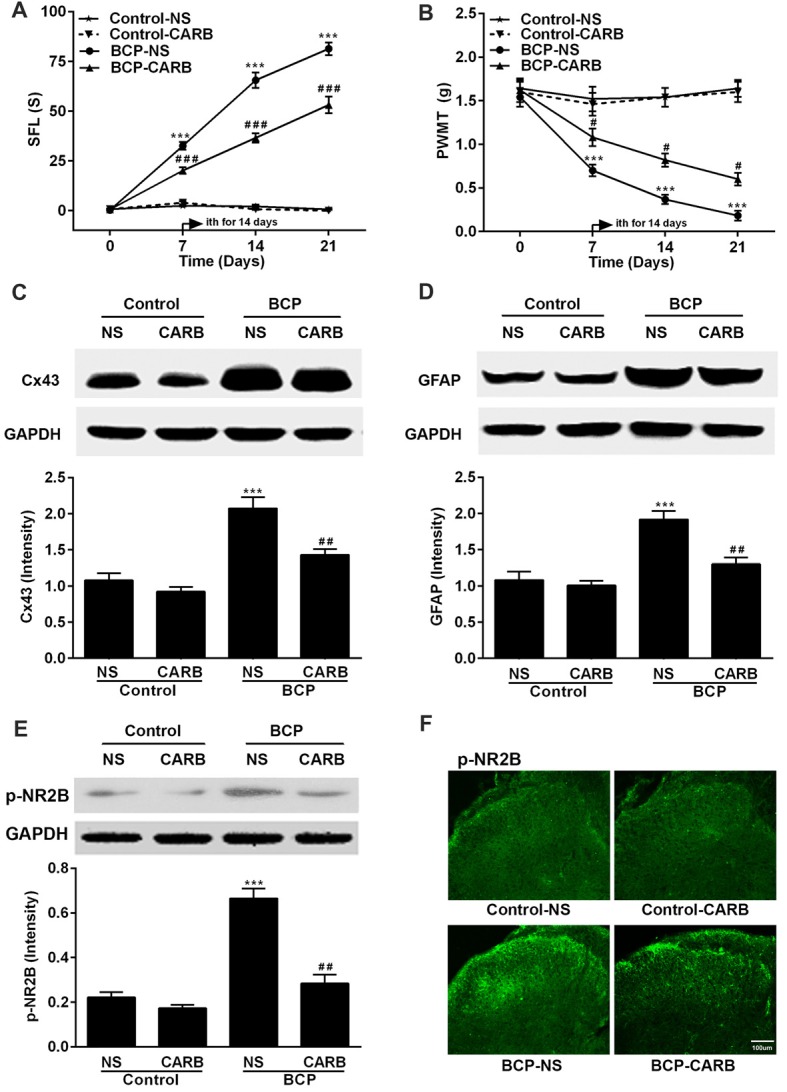
Effect of carbenoxolone (CARB) on BCP and the expressions of Cx43, GFAP and p-NR2B in the SDH in normal and BCP mice. **(A)** SFL test in control and BCP mice with or without CARB treatment. **(B)** Mechanical allodynia (PWMT) test in control and BCP mice with or without CARB treatment. **(C)** The expression of Cx43 in the SDH in control and BCP mice with or without CARB treatment by Western blot analysis. **(D)** The expression of GFAP in the SDH in control and BCP mice with or without CARB treatment by Western blot analysis. **(E)** The expression of p-NR2B in the SDH in control and BCP mice with or without CARB treatment by Western blot analysis; and **(F)** The expression of p-NR2B in the SDH in control and BCP mice with or without CARB treatment by immunofluorescence analysis (Bar = 100 μm). CARB was administrated at 1 mg/kg by intrathecal (ith) daily for consecutive 14 days (on day 7–20). SFL and PWMT tests were performed on day 0 (control), 7, 14 and 21 with 10 mice for each group (*n* = 10) and the expressions of Cx43, GFAP, and p-NR2B were on day 21 with five mice for each group (*n* = 5) after cell inoculation. GAPDH was used as a loading control for Western blot analysis. Data were presented as mean ± SE. ***p < 0.001 vs. control mice; ^#^*p* < 0.05, ^##^*p* < 0.01, or ^###^*p* < 0.001 vs. BCP mice without CARB treatment.

Mice were treated with CARB (1 mg/kg, ith) once daily for 14 days on day 7–20 after cell inoculation. CARB did not affect the pain threshold of control mice. However, CARB significantly suppressed both spontaneous pain (*p* < 0.001) and mechanical allodynia (*p* < 0.05) in the ipsilateral hind paws of BCP mice over time compared BCP mice treated with NS. BCP mice treated with NS exhibited remarkable pain hypersensitivity compared with that of control mice treated with NS (*p* < 0.001; Figures 4A,B). Meanwhile, the expressions of Cx43, GFAP and p-NR2B were significantly higher in BCP mice treated with NS than that of control mice treated with NS (*p* < 0.001) on day 21 after cell inoculation by Western blotting analysis. However, CARB markedly inhibited the expressions of spinal Cx43, GFAP and p-NR2B in BCP mice, compared to that of BCP mice treated with NS (*p* < 0.01; Figures 4C–E). Furthermore, treatment of CARB or NS did not affect the expression of p-NR2B in control mice but the expression of p-NR2B was significantly increased in BCP mice treated with NS by immunostaining analysis. Interestingly, CARB could significantly inhibit the expression of p-NR2B compared to BCP mice treated with NS (Figure 4F).

### MK801 Attenuates BCP and Inhibits the Expressions of p-NR2B and Cx43 in the SDH in BCP Mice

**Figure 5:**
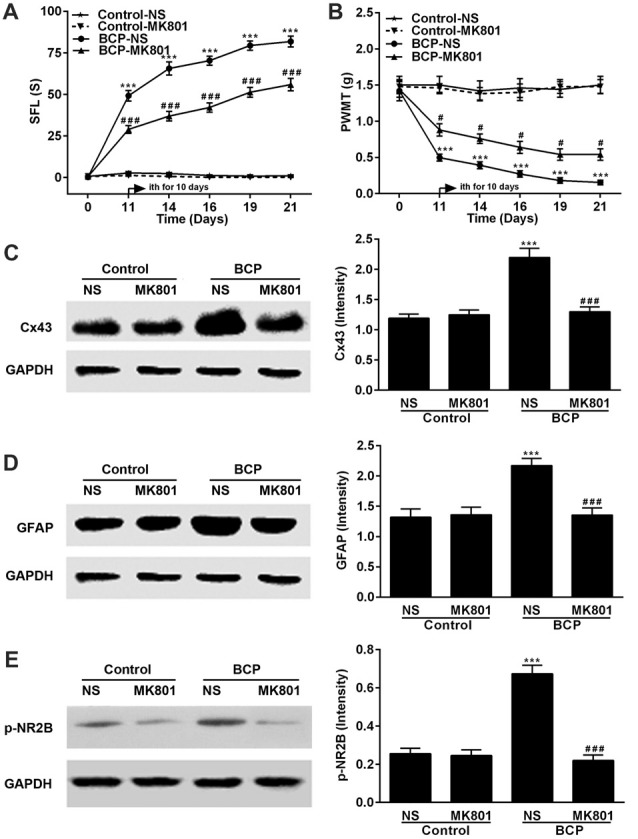
Effect of MK801 on BCP and the expressions of Cx43, GFAP and p-NR2B in the SDH in normal and BCP mice. **(A)** SFL test in control and BCP mice with or without MK801 treatment; **(B)** Mechanical allodynia (PWMT) test in control and BCP mice with or without MK801 treatment; **(C)** The expression of Cx43 in the SDH in control and BCP mice with or without MK801 treatment by Western blot analysis. **(D)** The expression of GFAP in the SDH in control and BCP mice with or without MK801 treatment by Western blot analysis; and **(E)** the expression of p-NR2B in the SDH in control and BCP mice with or without MK801 treatment by Western blot analysis. MK801 was administrated at 0.136 mg/kg by ith once day (daily) for consecutive 10 days (on day 11–20). SFL and PWMT tests were performed on day 0 (control), 7, 14 and 21 with 10 mice for each group (*n* = 10) and the expressions of Cx43, GFAP and p-NR2B were on day 21 with five mice for each group (*n* = 5) after cell inoculation. GAPDH was used as a loading control for Western blot analysis. Data were presented as mean ± SE. ***p < 0.001 vs. control mice; ^#^*p* < 0.05 or ^###^*p* < 0.001 vs. BCP mice without MK801 treatment.

Mice were treated with MK801 (0.136 mg/kg, ith) once daily for 10 days on day 11–20 after cell inoculation. MK801 significantly attenuated both spontaneous pain (*p* < 0.001) and mechanical allodynia (*p* < 0.05) in the ipsilateral hind paws of BCP mice over time compared to that of BCP mice treated with NS. The BCP mice treated with NS showed remarkable pain hypersensitivity compared to that of control mice treatment (*p* < 0.001) and MK801 did not affect the pain threshold of control mice (Figures 5A,B). Furthermore, the expressions of p-NR2B, Cx43 and GFAP in BCP mice treated with NS were remarkably higher than that of control mice treated with NS (*p* < 0.001) and MK801 or NS did not affect their expression levels in control mice on day 21 after cell inoculation by Western blotting analysis. However, MK801 markedly inhibited the expressions of p-NR2B, Cx43 and GFAP in BCP mice compared to that of BCP mice treated with NS (*p* < 0.001; Figures 5C,D).

### Co-localization of Cx43 and p-NR2B in the SDH

**Figure 6:**
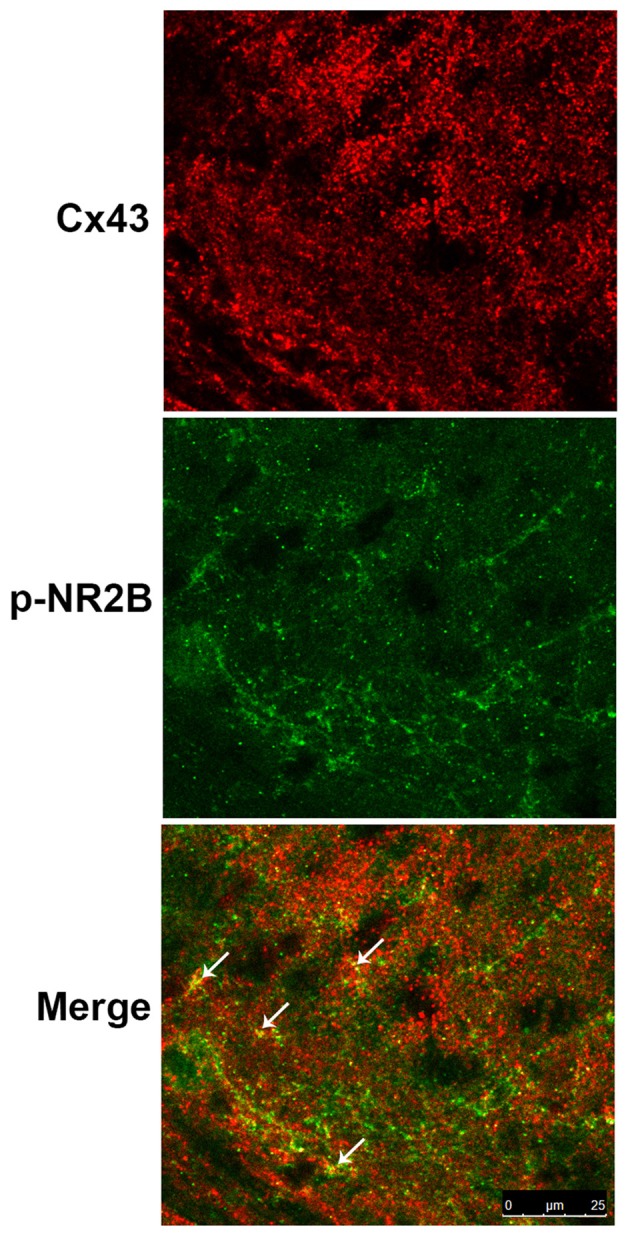
Representative confocal images of co-localization of Cx43 and p-NR2B in the SDH of BCP mice by immunofluorescence analysis. White arrows indicated the yellow dots as co-localization of Cx43 and p-NR2B. Tissues of spinal cord were collected from BCP mice on day 21 after cell inoculation. Bar = 25 μm. There were five mice used for each group (*n* = 5).

Study showed that Cx43 is primarily expressed in astrocytes but not in neurons while NR2B is expressed in both astrocytes and neurons (Liu et al., 2013). Therefore, we examined the expressions of Cx43 and p-NR2B in the spinal cord sections of BCP mice by double staining with confocal images and the results showed that Cx43 was partially co-localized with p-NR2B in the SDH in BCP mice (Figure 6), suggesting that Cx43 may have a close relationship with p-NR2B in astrocytes in BCP mice.

## Discussion

In the present study, our results demonstrated that the development and maintenance of BCP were related to the upregulated expressions of Cx43, GFAP and p-NR2B in the SDH and CARB (a GJ/hemichannel blocker) and MK801 (a NMDARs antagonist) could significantly alleviate BCP via inhibiting the expressions of Cx43, GFAP and p-NR2B in mice. Cx43 may have a close relationship with p-NR2B in astrocytes in BCP mice. The findings suggested that the combination action of Cx43 and p-NR2B in astrocytes of the SDH may contribute to the development and maintenance of BCP. These findings may provide a potential strategy for the clinical treatment of BCP.

BCP remains a challenge for clinicians because the underlying mechanisms of pain are still elusive (van den Beuken-van Everdingen et al., 2007). The animal models of BCP with various tumor cells (LLC, Walker 256 breast cancer, prostate cancer, and osteosarcoma) inoculation provide an experimental basis for the study of BCP (Currie et al., 2013). Our present study demonstrated that inoculation of LLC cells into the femur of C57BL/6 mice induced progressive SFL behaviors and mechanical allodynia in a time-dependent manner (Figures 1B,C). Moreover, bone destruction and tumor infiltration induced by LLC cells were confirmed by X-ray and pathohistological examination (Figures 1D,E). These results indicate that the mouse model of BCP was established by intra-femural inoculation of LLC cells and well mimicked the characters of BCP.

Connexins are the core proteins of GJs and hemichannels, and there are at least 20 connexins to constitute different types of GJs or hemichannels in mammals (Bennett et al., 2012; Vicario et al., 2017b). Cx43, one of the most abundant connexins in the CNS, is a main GJ/hemichannel forming protein mainly expressed by astrocytes (Chew et al., 2010; Vicario et al., 2017b). In the present study, we confirmed the observation that Cx43 was predominantly expressed in astrocytes, but not in neurons or in microglial cells by confocal images of spinal cord sections. The finding is in agreement with previous studies (Shen et al., 2014; Hang et al., 2016). However, it was reported that Cx43 was also found at the contact points between radial fibers of radial glia (the neuronal stem cells of the embryonic cerebral cortex) and migrating neurons, in endothelial cells, ependymal cells and tanycytes, developing neurons and activated microglial cells in the CNS (Elias et al., 2007; Chew et al., 2010; Salmina et al., 2014). Cultured rat and mouse microglia were found to express Cx43 after treatment of interferon (IFN-γ) plus TNF-α (Eugenin et al., 2001), which is inconsistent with the present study. The possible explanation may be due to different experimental condition and intervention method between *in vivo* and *in vitro*.

It was reported that the expression level of p-Cx43 (a phosphorylated form of Cx43 at the site of serine 368) was significantly upregulated accompanied by mechanical allodynia in BCP rats (Hang et al., 2016). In contrast, several studies have shown that the function of GJ was influenced by Cx43 phosphorylation (Quesseveur et al., 2015), and increase of p-Cx43 at serine 368 was correlated with down-regulation of GJ function (Palatinus and Gourdie, 2016). Moreover, our recent study showed that the expressions of both Cx43 and p-Cx43 were markedly suppressed but the ratio of p-Cx43/Total-Cx43, which was used to assess the relative expression level of p-Cx43, was not significantly changed by the treatment of ^43^Gap26 (a selective Cx43 mimetic peptide) in BCP mice, indicating that high expression of Cx43 but not p-Cx43 influenced the process of BCP (Li et al., 2017). Because of the controversial role of p-Cx43 on GJ function in BCP, we selected Cx43 rather than p-Cx43 for the present study.

CARB, a water soluble substance from medicinal licorice, is the succinyl ester of glycyrrhetinic acid and widely used in the treatment of gastric ulcer, polyarthritis and rheumatoid arthritis clinically (Azarashvili et al., 2014). Among the GJ inhibitors, CARB was also used as an effective Cx43 blocker for study of the function of Cx43 GJs and hemichannels (Suzuki et al., 2014; Wang et al., 2014; Li et al., 2016; Cheng et al., 2017; Szilvásy-Szabó et al., 2017; Vicario et al., 2017a). Furthermore, ith injection of CARB could alleviate mechanical allodynia in the mouse model of neuropathic pain more rapidly (less than 0.5 h) and persistent (more than 5 h) than that of ^43^Gap26, so making CARB more suitable for clinical therapy (Chen et al., 2014; Choi et al., 2016). Therefore, we used CARB instead of ^43^Gap26 to investigate the mechanisms of BCP in mice in the present study.

Activated astrocytes are deeply involved in the various condition of hyperalgesia induced by a variety of pathogenies including inflammation (Yamamoto et al., 2013), drugs (Liu et al., 2016) and spinal cord injury (Watson et al., 2014). GFAP is a cytoskeletal intermediate filament protein selectively expressed by matured astrocytes (Middeldorp and Hol, 2011). Moreover, it is one of the ideal markers for activated astrocytes (Zhang et al., 2017). Numerous studies have confirmed that activated astrocytes in the spinal cord exert notable action in pain hypersensitivity and are strongly implicated in the pathogenesis of BCP (Ren et al., 2012; Jiang et al., 2017). In the present study, we also found that the expression level of spinal GFAP was remarkably upregulated over time and could be suppressed by CARB or MK801 in BCP mice (Figures 4, 5), suggesting that the activation of astrocytes in the SDH was responsible for the development and maintenance of BCP in mice.

NMDARs are a type of the ionotropic glutamate receptors widespread in the mammalian CNS. They have been found to play critical roles in variant physiological functions and pathological situations including synaptic plasticity, neuronal development, learning, memory and chronic pain (Mony et al., 2009; Wu and Zhuo, 2009). Among NMDA receptor subunits, the NR2B receptors, especially tyrosine 1472 phosphorylated NR2B (p-NR2B) are particularly crucial for nociception and synaptic plasticity (David et al., 2014; Sarantis et al., 2015). Protein phosphorylation is one of the major regulation mechanisms for the function of receptors. It was reported that p-NR2B, but not total NR2B, contributed to inflammatory pain process (Bu et al., 2015). The important roles of NR2B phosphorylation in central sensitization, peripheral sensitization and various pain conditions have been generally accepted for a long time (Li et al., 2011; Xu et al., 2012; Peng et al., 2013; Liang et al., 2017). In the present study, the expression of p-NR2B in the SDH of BCP mice was markedly increased in a time-dependent manner accompanied by the severe pain hypersensitivity after the inoculation of LLC cells into the femur, which is consistent with previous reports (Liu et al., 2014; Sun et al., 2016), indicating the important role of p-NR2B in the development and maintenance of BCP.

NMDARs are generally considered as neuron-specific, however, growing evidence has indicated that NMDARs are also expressed in astrocytes. Functional NR2B was found in astrocytes co-cultured with neurons after ischemia or anoxia treatment (Krebs et al., 2003). Cortical astrocytes of mice in primary cultures expressed differentially mRNA of NMDARs subunits (NR1, NR2A and NR2B) in development and pathological conditions of ischemia and post-ischemia without the existence of neurons (Zhou et al., 2010). Moreover, human primary astrocytes were detected for the functional presence of all seven known NMDARs subunits, including NR1, NR2 (A, B, C, D), NR3 (A, B). Further studies revealed that glutamate could activate astrocytic NMDARs to trigger Ca^2+^ influx into astrocyte (Lee et al., 2010). It was reported that NR2B, but not NR1, was expressed in both astrocytes and microglial cells in the SDH of normal and BCP rats (Liu et al., 2013).

In the present study, we found that p-NR2B was parallelly increased and co-localized with Cx43 in the SDH in BCP mice, which further evidenced the presence of functional NR2B in astrocytes and the essential roles of Cx43 and p-NR2B in BCP. Notably, as a GJ/hemichannel blocker, CARB not only markedly inhibited the expressions of Cx43 and GFAP but also decreased the expression of p-NR2B with alleviated spontaneous pain and mechanical allodynia in BCP mice. Interestingly, MK801 (a NMDAR antagonist) exhibited the same effects as CARB, which it not only decreased the expression of p-NR2B, but also suppressed the expressions of Cx43 and GFAP in the SDH with pain attenuation in BCP mice. These findings suggested that Cx43 and p-NR2B may enhance the activation of each other in BCP mice. Therefore, we speculated that Cx43 and p-NR2B in astrocytes of the SDH may synergistically contribute to the development and maintenance of BCP.

The mechanisms of CARB preventing the increase of Cx43, GFAP and p-NR2B in BCP mice are very complex. We suspect that CARB may bind to Cx43 GJ channels and induce conformational modification to decrease the expression level of Cx43, ultimately contributing to suppress the activation of spinal astrocytes (evidenced by the expression of GFAP), which is dependent on the intact function of GJs (Yoon et al., 2013), and to reduce the subsequent phosphorylation of NR2B in the SDH in BCP. Moreover, MK801 decreased the expressions of p-NR2B, Cx43 and GFAP in the SDH in BCP mice, suggesting that the activation of NMDARs was involved in the upregulated expression of Cx43 and the astrocytes activation. Up to date, the underlying mechanisms of the inhibitory actions of CARB and MK801 on Cx43, GFAP and p-NR2B in spinal astrocytes in BCP mice remain unclear, and are needed to be further investigated.

When BCP occurs, the pain signaling is transmitted from stimulated peripheral nociceptive receptors to the SDH, which lead to abundant release of excitatory neurotransmitter (glutamate) and subsequent activation of Ca^2+^ influx mediated by NMDARs and diverse Ca^2+^-dependent signals (Morales et al., 2012; Liu et al., 2015). The increased Ca^2+^ influx via NMDARs induces synaptic plasticity, which contributes to the NMDAR (specifically NR2B subunit)-dependent central sensitization (Naoki et al., 2005; Takeuchi et al., 2011; Cibert-Goton et al., 2013). GJ and hemichannel are two types of Cx43 channel, and the increase of levels was found to depend on the intracellular Ca^2+^ signal increase (Salas et al., 2015). Glutamate, as an important neurotransmitter and gliotransmitter, can activate Cx43 hemichannel and increase the activity of GJ and hemichannel in astrocytes (Orellana et al., 2013). The rise in intracellular calcium ion concentration ([Ca^2+^]_i)_ or decrease in extracellular calcium ion concentration ([Ca^2+^]_e_) occurred during synaptic activity can stimulate hemichannel opening by multiple intermediate signal pathways (De Vuyst et al., 2009; Torres et al., 2012). In turn, opened Cx43 hemichannels trigger the release of glutamate into the extracellular space and synaptic cleft activating presynaptic and postsynaptic NMDARs, thus resulting in the widespread propagation of synaptic activity in the CNS (Chever et al., 2014; Ferrari et al., 2014; Orellana and Stehberg, 2014).

Recent evidence suggests that astrocytes in the spinal cord enwrap pre-synaptic and post-synaptic neurons to constitute a number of functional elements termed “tripartite synapse”, which lead to bidirectional communication and modulation between astrocytes and neurons in networking activity of CNS (Charveriat et al., 2017). Both Cx43 and NMDARs are responsible for the function of tripartite synapse through glutamate release and activation of Ca^2+^ signaling (Kekesi et al., 2015; Zhang et al., 2016). Thereby, there may be a glutamate/Ca^2+^-dependent positive feedback loop by Cx43/p-NR2B-mediated astrocyte-astrocyte, astrocyte-neuron and neuron-astrocyte signaling in the SDH in the development of BCP. The relationship between Cx43 and astrocytic p-NR2B may represent a novel pathogenesis underlying the development and maintenance of BCP in mice. However, additional studies with *in vitro* and *in vivo* experiments are still required to further elucidate the important roles of Cx43 and astrocytic p-NR2B and associated mechanisms in BCP.

## Conclusion

The present study is the first to evidence that inhibition of Cx43 and p-NR2B in spinal astrocytes could attenuate BCP in mice and the combination action of Cx43 and p-NR2B in the astrocytes of the SDH may contribute to the development and maintenance of BCP in mice. The spinal Cx43/p-NR2B pathway may be an important pathway for BCP. The relationship of spinal Cx43 and astrocytic p-NR2B may play an important role in astrocyte-astrocyte signaling in the development and maintenance of BCP in mice. These results may provide the scientific rationale for better understanding of the molecular and cellular mechanisms associated with BCP, and a potential analgesic strategy for the treatment of BCP clinically.

## Author Contributions

DH and LW designed and supervised the research. HYang, HYan and JL performed the experiments. HYang, JL, SC, BH and XL analyzed the data. HYang and HYan wrote the draft and SC wrote the final version of the manuscript. All authors approved the final manuscript for publication.

## Conflict of Interest Statement

The authors declare that the research was conducted in the absence of any commercial or financial relationships that could be construed as a potential conflict of interest.

## Abbreviations

ANOVA: analysis of variance
BCP: bone cancer pain
CARB: carbenoxolone
CNS: central nervous system
Cx: connexin
DMEM: Dulbecco’s modified Eagle medium
EDTA: Ethylenediaminetetraacetic Acid
FBS: fetal bovine serum
GAPDH: glyceraldehyde-3-phosphate dehydrogenase
GFAP: glial fibrillary acidic protein
GJ: gap junction
HBSS: Hank’s balanced salt solution
HE: hematoxylin and eosin
HRP: horseradish peroxidase
i.p.: intraperitoneal
ith: intrathecal
LLC: Lewis lung carcinoma
NMDARs: N-methyl-D-aspartate receptors
NR2B: N-methyl-D-aspartate receptor 2B subunit
NS: normal saline
PBS: phosphate buffer saline
p-Cx43: phosphorylated Cx43
p-NR2B: phosphorylated NR2B
PVDF: polyvinylidene difluoride filters
PWMT: paw withdrawal mechanical threshold
SDH: spinal dorsal horn
SE: standard error
SFL: spontaneous foot-lifting
SPF: specific-pathogen-free
vs: versus.
